# Effectiveness and safety of 3 different traditional Chinese therapies for asthma in minors: A protocol for systematic review and network meta-analysis

**DOI:** 10.1097/MD.0000000000023021

**Published:** 2020-11-20

**Authors:** Shi Fan Yan, Ting Yu, Fu Sheng Li, Yong Huang, Mao Hong Wang

**Affiliations:** a Jiangxi University of Traditional Chinese Medicine; b Affiliated Hospital of Jiangxi University of Traditional Chinese Medicine, Nanchang, China.

**Keywords:** asthma, network meta-analysis, protocol

## Abstract

**Introduction:**

Traditional Chinese medicine has been widely used in the treatment of asthma, but currently there has been no comprehensive evaluation on the efficacy and safety of different traditional Chinese medicine therapies, based on network meta-analysis (NMA).

**Methods and analysis:**

The retrieval strategy was strictly followed in the English databases PubMed, Embase, Cochrane Library, and Chinese databases: CNKI and Wanfang. The retrieval time was limited from the beginning of each database construction to September 12, 2020. Meanwhile, in order to prevent data missing, we compared the previous meta-analysis to determine the final inclusion results. The main indexes were Spirometry, the secondary indexes were Asthma Quality of Life Questionnaire and adverse events. Methodological quality of articles was assessed using the Cochrane Collaborative tool, and evidence quality was assessed using the Recommended Scoring, Development, and Evaluation tool. Completed The NMA using Stata statistical software (Version 14.0, Stata Corporation, College Station, TX). The Cochrane Collaboration's Tool was used to evaluate the methodological quality of articles, the Grading of Recommendations Assessment, Development, and Evaluation instrument was used to evaluate the quality of evidence. NMA was completed by using Stata Statistical Software (Version 14.0, Stata Corporation, College Station, TX).

**Results:**

The study will rank 3 traditional Chinese medicine treatments for asthma.

**Conclusion:**

This study is the first time to use NMA to compare the efficacy and safety of traditional Chinese medicine for the treatment of asthma, which will provide ideas and methods for the clinical treatment for asthma.

**INPLASY registration number:**

INPLASY202090052

## 1 Introduction

Asthma, or bronchial asthma, is a common chronic respiratory disease characterized by chronic inflammation of the airway.^[1,2]^ Its pathogenesis is mainly related to airway inflammation, airway hyperreactivity, neural mechanism, allergy, etc. Heredity and environment are the main causes of asthma, and the main inducing factors are dust, smoke, climate, exercise, respiratory tract infection, mental, and psychological factors, trace element deficiency, drugs, etc.^[3]^ clinical manifestations are often repeated wheezing, shortness of breath, chest tightness, cough, and other symptoms, often at night and in the early morning attack or worse.^[4]^ According to epidemiological survey, there are about 334 million people with asthma in the world, which is more than 5% of the total population. However, among children, the prevalence rate of asthma has significantly increased, and about 14% of children have asthma, which has become the most common non-communicable disease.^[1,5]^ asthma, on the 1 hand, affects the children's life and study, and on the other hand, aggravates the social and economic burden.^[6–9]^

Because the cause of the age, the treatment of children compared to adults have more restrictions, in addition to the daily life away from the inducing factors, drug therapy is an extremely important part of now is mainly used in the treatment of asthma drugs beta Agonists, Inhaled steroids, Systemic steroids, Anticholinergics, Leukotriene pathway modifiers, Theophylline Anti - IgE, these drugs are more or less have some side effects, Such as Tremors, Dysphonia, Hyperglycemia, Dry expressions using, Elevated liver enzymes, Gastritis, Anaphylaxis, and so on, and even some drugs will delay the growth of children.^[3]^

Based on the above problems, we should pay attention to finding a treatment method suitable for children. Traditional Chinese medicine therapy has a history of thousands of years in China, among which acupuncture, acupoint application, moxibustion and other treatment methods are widely used in asthma, and some studies have proved that the intervention of asthma is quite effective, and no obvious side effects have been found for the time being.^[10–13]^ However, for different traditional Chinese medicine (TCM) therapies, there are differences in clinical efficacy, and there is still no consensus on which method we should give priority to. To solve this problem, we used network meta analysis to rank the effectiveness of various treatment measures and determine the final result by comparing sucre values.

## 2 Methods and analyses

### 2.1 Design

Systematic review and network meta-analysis (NMA).

### 2.2 Patient and public involvement

This study is a secondary literature study and does not involve clinical patients or the general public.

### 2.3 Eligibility criteria

#### 2.3.1 Types of studies

The included articles determine the credibility of the final results. Therefore, we only select randomized controlled trials (RCTs) of traditional Chinese medicine therapy for asthma, and the intervention measures are only limited to acupuncture, moxibustion, acupoint sticking or blank control, excluding Chinese herbal medicine, massage, school, and other traditional Chinese medicine therapies.

#### 2.3.2 Type of participant

For all minors (5–18 years old) diagnosed with Asthma, the diagnostic criteria follow Guidelines for the Diagnosis and Management of Asthma (EPR-3)^[14]^, and there are no requirements for country, region, race, or gender.

#### 2.3.3 Interventions

The intervention measures were only included in acupuncture, moxibustion, and acupoint sticking. Among them, acupuncture tools could only choose millineedle, but there was no requirement for acupuncture needle application. Different moxibustion techniques were selected, including ginger moxibustion, heat-sensitive moxibustion, wheat grain moxibustion and mild moxibustion. There is no restriction on the use of Chinese herbs in acupoint application. Trials in multiple trial groups will also be included if all 3 of the above interventions are included.

#### 2.3.4 Types of outcome measurements

##### 2.3.4.1 Primary outcome

Spirometry^[15]^: This can help us better assess the condition of the lungs, we use 4 main indicators: Forced vital capacity, forced expiratory volume in 1 s, the forced expiratory volume in 1 s/forced vital capacity ratio, and peak expiratory flow.

##### 2.3.4.2 Secondary outcomes

1) Asthma Quality of Life Questionnaire^[16]^: It mainly includes 5 aspects: activity restriction, asthma symptoms, psychological status, response to stimuli, and concern for one's own health. There are a total of 35 questions, with a score of 1 to 7 for each question. Higher scores mean better results
2) The incidence rate of adverse events.

#### 2.3.5 Exclusion criteria

1) Exclusion of reviews, animal experiments, case reports, and non-RCTs.
2) Exclude the trials of TCM therapy combined with other therapeutic means,
3) Exclude contrast between different acupuncture and moxibustion.
4) Exclude tests with severe data loss or errors.

### 2.4 Literature search

Comprehensive searches of RCTs on traditional Chinese medicine therapy for asthma were conducted in 3 English databases of PubMed, Cochrane Library, Embase and 2 Chinese databases of CNKI and Wanfang, and the time of index was from their inceptions to September 10, 2020 for each database. The retrieval strategy of PubMed is shown in Table 1. Two team members (YT and YSF) independently searched the article according to the retrieval strategy, they also exported the citations.

**Table 1:** Search strategy used in PubMed database.

Number	Search items
#1	Randomized controlled trial [pt]
#2	Controlled clinical trial [pt]
#3	Randomized [tiab]
#4	Clinical trials as topic [mesh: noexp]
#5	Randomly [tiab]
#6	Trial [ti]
#7	Or/#1–#6
#8	Asthma[MeSH]
#9	Asthma, occupational [MeSH]
#10	Asthmas or bronchial asthma or asthma, bronchial or asthma, occupational [all fields)
#11	Or/#8–#10
#12	Acupuncture [MeSH]
#13	Acupuncture therapy [MeSH]
#14	Previous indexing or pharmacopuncture or acupuncture treatment or acupuncture treatments or treatment, acupuncture or therapy, acupuncture or pharmacoacupuncture treatment or treatment, pharmacoacupuncture or pharmacoacupuncture therapy or therapy, pharmacoacupuncture or acupotomy or acupotomies [all fields)
#15	Or/#12–#14
#16	Moxibustion [MeSH]
#17	Moxabustion [all fields)
#18	Or/#16–#17
#19	Acupuncture points [MeSH]
#20	Acupoint [all fields)
#21	Acupuncture points [all fields)
#22	Point, acupuncture [all fields)
#23	Or/#19–#22
#24	#15 or #18 or #23
#25	7 and #11 and #24

### 2.5 Data collection

#### 2.5.1 Selection of studies

**Figure 1:**
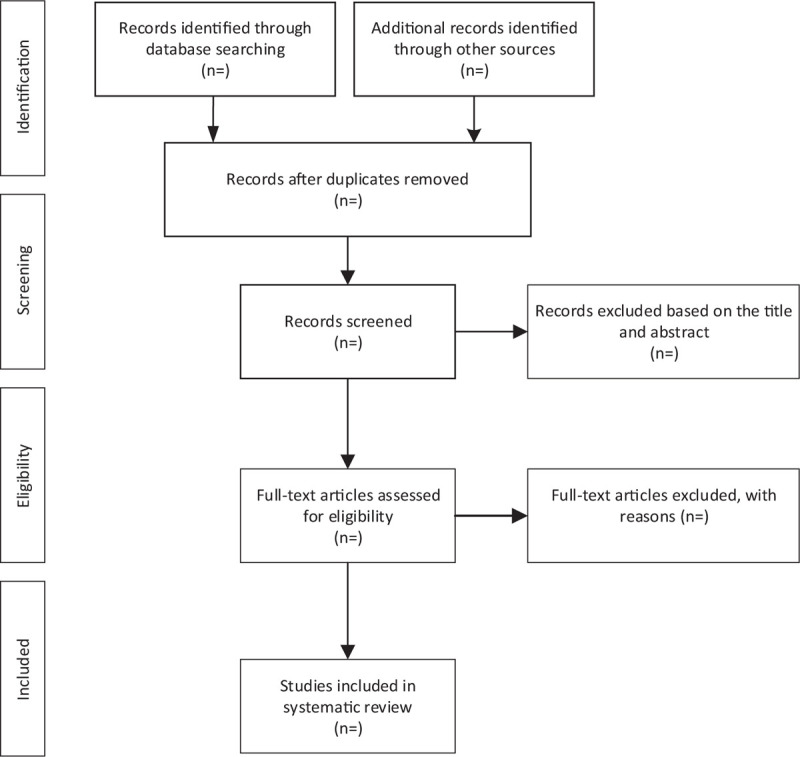
Flow diagram of study selection process.

For the convenience of management, we searched from 5 databases and imported titles into EndNote Software AQ8 (V.X9). Firstly, we used the software to remove duplicate articles, then 2 team members (YT and YSF) independently read the titles and abstractions, they deleted the literature that did not meet the requirements, and read the full text of the remained articles to decide the final inclusion of the experiment. After that, cross-checking to the results of both parties was conducted. If there is any disagreement, the decision would be made via group discussion. The entire process and results are shown in Figure 1.

#### 2.5.2 Data extraction and management

Microsoft Excel 2016 was used to establish information data extraction table, and pre-extraction was carried out to determine the feasibility of the table. Then 2 team members (YT and YSF) independently extracted the following information after training:

1) Basic information: Title, author, country, year, language, etc.
2) Baseline information: Gender, age, number of persons, country, diagnostic criteria, etc.
3) Methodological information: Grouping method, allocation concealment, blind method, result bias, etc.
4) Intervention measures: Treatment measures, treatment time, frequency, etc.
5) Results: Data of primary and secondary results.

After the work is completed, the results are cross-checked, if there are differences, a group discussion is conducted to determine the final result.

### 2.6 Assessment of risk of bias in included studies

The 2 authors (YT and YSF) evaluated the article methodology of inclusive trials independently, by the Cochrane collaboration “Bias risk” tool sequences generated from 6 aspects of allocation concealment, blind (or mask), incomplete data evaluation, evaluation reports, and other sources of bias selective results. Finally, for each items, we made ranking of “Low-risk bias”, “High-risk bias” and “Unclear” based on the Cochrane collaboration “bias risk” tool.^[17,18]^

### 2.7 Data analysis

#### 2.7.1 Management of lost data

If data is insufficient from the selected study, we will contact the author via email for the required data. If baseline and outcome data or other data are included, the mean and standard deviation of the change will be manually calculated according to the Cochrane.^[19]^

#### 2.7.2 Network map

In the network diagram, each dot represents an intervention; The larger dot area means the bigger population of the studied intervention; The line between the 2 dots represent that there is direct comparison to RCT studies among 2 interventions; The line thickness represents the numbers of direct comparison to RCT studies among 2 interventions.

#### 2.7.3 Transitivity and consistency assessment

Transitivity and consistency are the prerequisites for reticular meta-analysis. The transitivity was evaluated qualitatively from the perspective of methodology and was evaluated according to the PICO principle. Consistency was mainly to check local and overall consistency. Local consistency can be checked by loop consistency test (Higgins model). The global consistency test was verified by the corresponding inconsistency model according to different data.

#### 2.7.4 Assessment of heterogeneity

Heterogeneity tests for all included studies were performed by using Network prediction interval graph, then to study the relationship of the weighted mean difference at a 95% confidence interval and estimation zone (95%Prl) to invalid line, only when invalid line crosses perpendicularly to estimation zone but doesn’t to confidence interval, then means heterogeneity exists.^[20]^

#### 2.7.5 Pairwise meta-analysis

If there is a direct comparison between the experimental interventions included in the data (TCM versus TCM, TCM versus placebo), the Stata14.0 will be used for Pairwise meta-analysis based on a random-effects model.

#### 2.7.6 NMA

Two team members (YT and YSF) used statistical software - Stata (version 14.0, Stata Corporation, College Station, TX) for analysis. A random effects model was used for NMA to compare the variables between different interventions. By comparing Surface Under the Cumulative Ranking Curve, the optimum intervention measures were determined. The range of surface under the cumulative ranking curve is 0 to 100%, the higher of the cumulative ranking curve means the better of the efficacy.^[21]^

#### 2.7.7 Assessment of reporting biases

Funnel plots are used to detect publication bias. If the images are asymmetric, it indicates that there is publication bias.

#### 2.7.8 Subgroup analysis

If the analysis shows significant heterogeneity, then the root cause will be analyzed according to the PICOS principle, and the STATA 14.0 will be used for subgroup analysis.

#### 2.7.9 Grading the quality of evidence

According to the standards in the Grading of Recommendations Assessment Development and Evaluation system^[22]^, 2 team members evaluate the quality of the research and divide it into 4 levels of “high”, “medium”, “low” and “very low”, then the results will be exchanged. If there is any disagreement, the final option will be selected via group discussion.

### 2.8 Ethics and dissemination

The secondary literature study has no relationship to the personal data of the study, so the ethical approval is not required. Evaluation of the efficacy and safety of different traditional Chinese medicine therapies for allergic rhinitis may provide evidence for clinical treatment of this disease. The results of the study will be published in a peer-reviewed journal.

## Author contributions

WMH conceived this study. YT and YSF completed the project, search strategy, research selection, bias risk assessment, data extraction, data analysis and evidence quality assessment, LFS and HY assisted to the project revision and bias risk assessment, WMH assisted to the analysis and evidence quality assessment, YT wrote the original manuscript, YSF reviewed and edited the documents. All the authors approved the final project.

**Conceptualization:** Mao Hong Wang.

**Data curation:** Shi Fan Yan, ting yu, Mao Hong Wang.

**Formal analysis:** Shi Fan Yan, ting yu.

**Funding acquisition:** Yong Huang.

**Investigation:** Yong Huang.

**Methodology:** Shi Fan Yan, ting yu, Fu Sheng Li.

**Software:** Shi Fan Yan.

**Supervision:** Fu Sheng Li, Yong Huang, Mao Hong Wang.

**Visualization:** Fu Sheng Li, Yong Huang.

**Writing – original draft:** ting yu.

**Writing – review & editing:** Shi Fan Yan.
